# Doxycycline-Associated Hyperpigmentation: A Case Report and Literature Review

**DOI:** 10.7759/cureus.23754

**Published:** 2022-04-02

**Authors:** Antara Afrin, Philip R Cohen

**Affiliations:** 1 Dermatology, Michigan State University College of Human Medicine, East Lansing, USA; 2 Dermatology, University of California, Davis Medical Center, Sacramento, USA

**Keywords:** skin, scar, rash, photosensitivity, minocycline, hyperpigmentation, face, doxycycline, darkening, acne

## Abstract

Drug-induced hyperpigmentation is an adverse cutaneous effect; it has been associated with several systemic medications. A healthy 40-year-old man developed facial and dorsal hand hyperpigmentation within two weeks of beginning doxycycline monohydrate 100 milligrams twice daily for acne. Skin pigmentation significantly diminished at a follow-up evaluation two months after discontinuing the medication. Doxycycline-associated skin hyperpigmentation, albeit uncommon, has been described in 18 patients in the literature, including our patient. The demographics included 13 males and five females ranging in age from 11 to 87 years; eight of the patients were less than 50 years old and ten of the patients were over 60 years old. Doxycycline-associated hyperpigmentation frequently occurs on the face and can occur at the site of a previous scar. In most cases, doxycycline was discontinued with the resolution of hyperpigmentation.

## Introduction

Doxycycline is an antibiotic agent that belongs to the tetracycline class. Its mechanism of action involves reversibly binding to the 30S ribosomal subunit to prevent bacterial protein synthesis. It is commonly used for short-term therapy in the treatment of acute infections, such as ones caused by methicillin-resistant Staphylococcus aureus, and in the long-term management of acne vulgaris [1,2].

Hyperpigmentation is the darkening of the skin. There are several potential etiologies that can result in hyperpigmentation. They include melasma, vitamin deficiencies, and medications [3,4].

In patients with drug-associated hyperpigmentation, skin darkening can occur as the result of the drug alone or after sun exposure. Tetracyclines, and in particular minocycline, are among the most common agents associated with drug-induced pigmentation. Four types of mechanisms have been described in the literature in the pathogenesis of drug-induced pigmentation: accumulation of melanin, accumulation of the triggering medication, synthesis of special pigments, and deposition of iron [3-6].

A 40-year-old man developed cutaneous hyperpigmentation on his face and dorsal hands after beginning treatment with doxycycline for acne. His skin darkening resolved after the drug was discontinued. Clinical features of individuals with drug-associated skin hyperpigmentation, nail discoloration, or both after starting treatment with doxycycline are reviewed [7-16].

## Case presentation

A healthy 40-year-old Hispanic man presented for evaluation of adult acne. During his teenage years, he suffered from severe facial acne, which was resolved using topical and systemic antibiotic therapy. He also has a history of lichen simplex chronicus on his bilateral dorsal fingers, which had been successfully treated with fluocinonide 0.05% cream twice daily. He currently was not using any topical or systemic medications.

Cutaneous examination showed inflammatory papules on his forehead and bilateral malar cheeks. There were also acne scars on both cheeks. The treated areas of lichen simplex chronicus on the distal and proximal interphalangeal joints of his fingers had flattened.

His facial lesions were diagnosed as adult acne. Initial treatment included doxycycline monohydrate 100 milligrams twice daily. In addition, once-daily topical therapy with benzoyl peroxide 5% gel and clindamycin phosphate 1% solution was also applied.

He returned for a follow-up evaluation after six weeks. He was pleased that all his acneiform lesions had cleared after two weeks of therapy. However, after two weeks of treatment, he developed hyperpigmentation on his face and hands. He denied any sun exposure.

**Figure 1:**
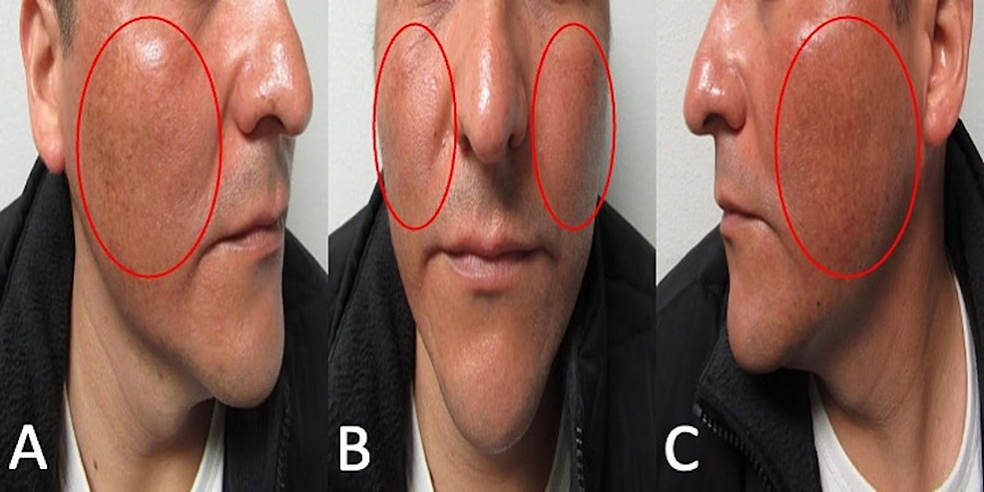
Clinical presentation of doxycycline-associated hyperpigmentation on the face Right-side view (A), frontal view (B), and left-side view (C) of the face of a 40-year-old man who developed skin darkening within two weeks of beginning doxycycline therapy; there was no history of sun exposure. The areas of hyperpigmentation (red ovals) are observed on the bilateral malar cheeks.

**Figure 2:**
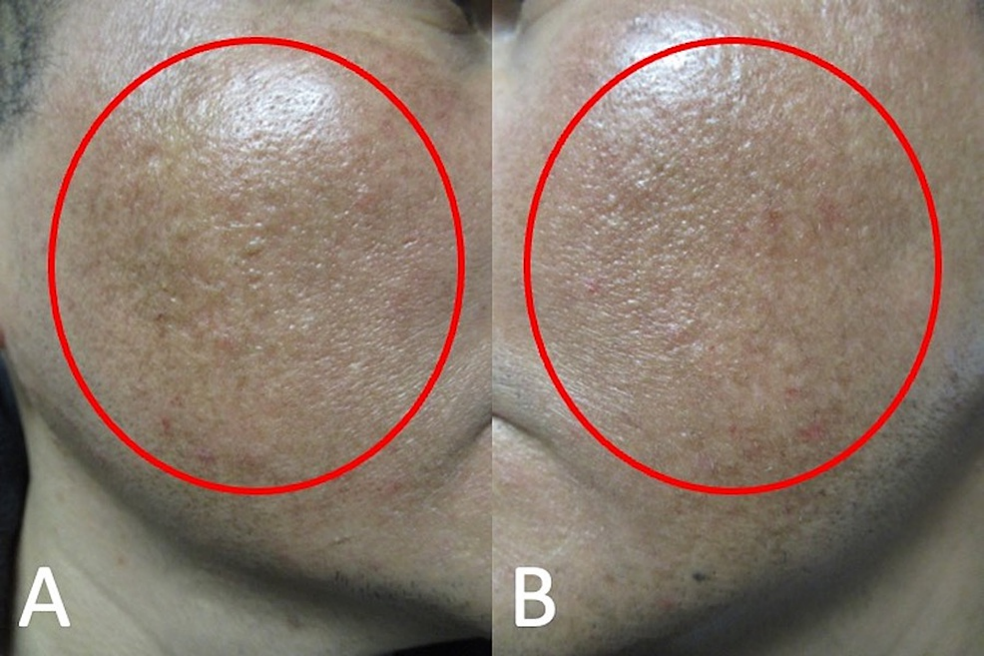
Cutaneous hyperpigmentation induced by doxycycline on areas of acne scars on malar cheeks Doxycycline-associated hyperpigmentation (red ovals) is in areas of old acne scars on right (A) and left (B) malar cheeks.

**Figure 3:**
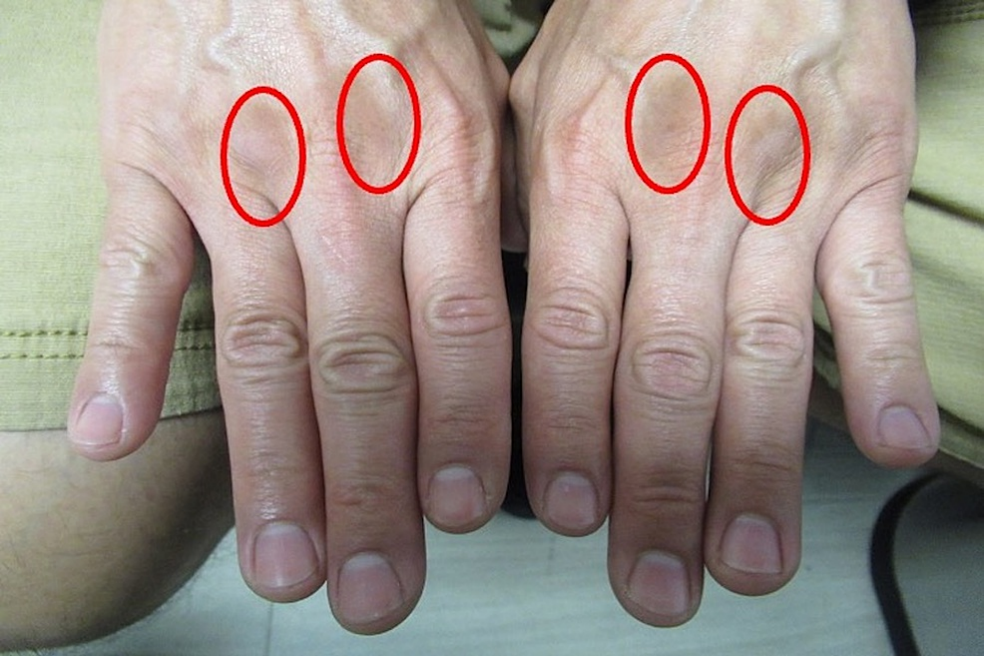
Doxycycline-associated skin pigmentation on dorsal hands Hyperpigmentation from doxycycline (red ovals) developed between not only the second and third, but also the third and fourth metacarpophalangeal joints on both hands.

Cutaneous examination showed hyperpigmentation of the skin on his forehead, nose, bilateral temple regions, and both malar cheeks extending to his jaw (Figures 1A-1C). Skin hyperpigmentation was found in the areas of acne scars on both of his malar cheeks (Figures 2A, 2B). In addition, there was hyperpigmentation on his bilateral dorsal hands between not only the second and third but also the third and fourth metacarpophalangeal joints (Figure 3). There was no hyperpigmentation of the mucosal membranes. Additionally, there was no subungual or nail pigmentation.

The correlation of the patient’s clinical history and skin findings established a diagnosis of doxycycline-associated hyperpigmentation. Initial management included stopping the doxycycline monohydrate. To maintain his acne clearing, he was also prescribed erythromycin ethylsuccinate 500 milligrams twice daily. He continued to use the benzoyl peroxide 5% gel and clindamycin phosphate 1% solution once daily.

**Figure 4:**
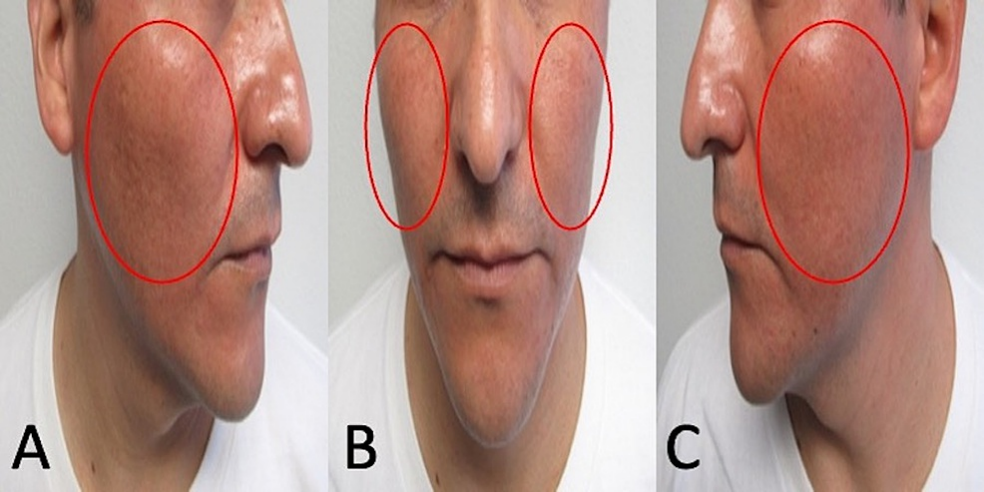
Significant clinical resolution of doxycycline-association hyperpigmentation on the face Right-side view (A), frontal view (B), and left-side view (C) of the face show significant fading of hyperpigmentation on the bilateral malar cheeks (red ovals) within two months after stopping doxycycline.

**Figure 5:**
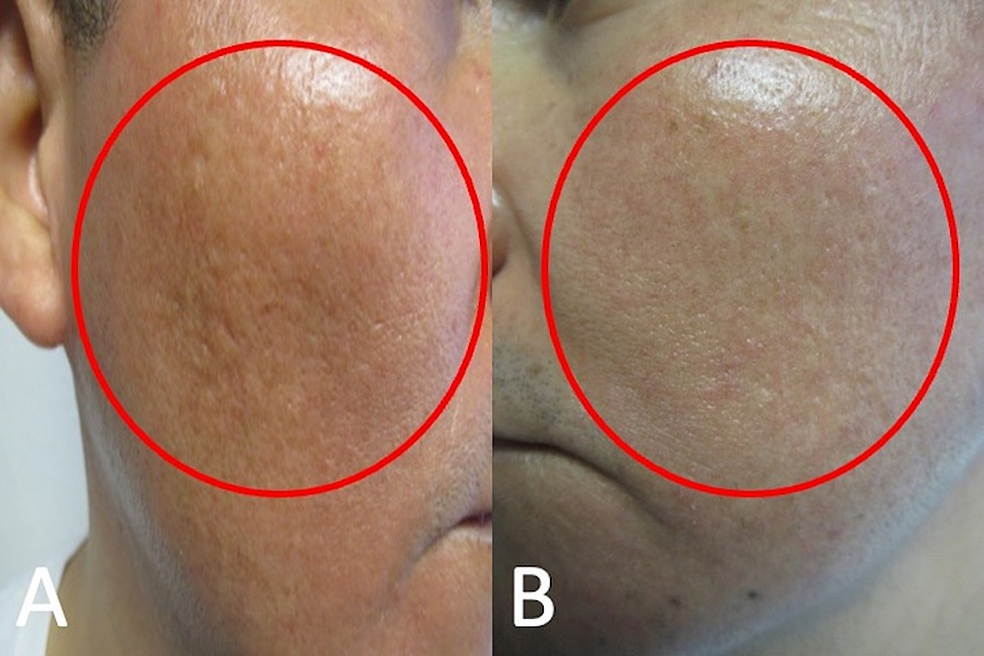
Improvement of doxycycline-related hyperpigmentation in areas of old acne scars Right-side view (A) and left-side view (B) of malar cheeks demonstrate regression of doxycycline-associated hyperpigmentation (red ovals).

**Figure 6:**
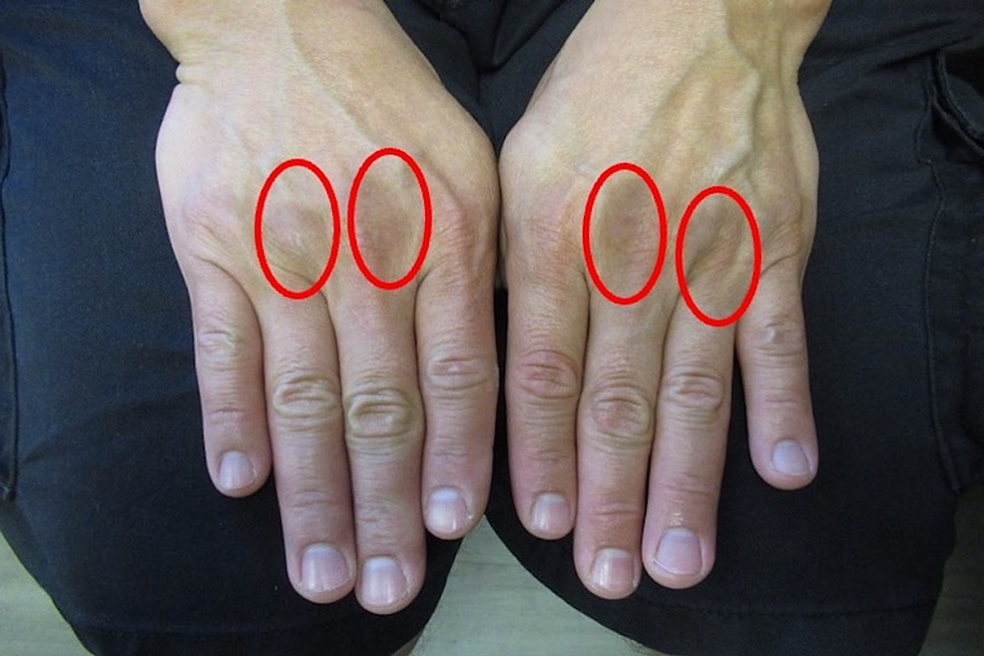
Improvement of doxycycline-associated skin pigmentation on dorsal hands Areas of decreased hyperpigmentation (red ovals) are observed between not only the second and third but also the third and fourth metacarpophalangeal joints on both hands after stopping doxycycline.

He returned for evaluation after two months. There had been significant lightening of the skin pigmentation on his face (Figures 4A-4C, 5A-5B) and dorsal hands (Figure 6). His current therapy was continued, and he planned to return for follow up in four months.

## Discussion

Doxycycline can be associated with various side effects. These can be systemic such as such headaches, pseudotumor cerebri, and hand tremors. Cutaneous adverse events associated with doxycycline have also been described, including morbilliform exanthem, photosensitivity, and rarely, skin and nail hyperpigmentation [7-18].

Drug-induced hyperpigmentation has been associated with several classes of medications [4]. Tetracyclines, and in particular minocycline, not only cause cutaneous pigmentation but also mucosal (including the sclera and tongue) and subungual pigmentation [5,6]. However, albeit less common, doxycycline can cause hyperpigmentation [7-16].

Minocycline-induced skin hyperpigmentation has been described in three clinical variants. The first type is blue-black pigmentation occurring within scars. The second is the same type of pigmentation occurring on normal skin. The third type is pigmentation in areas that are sun-exposed and appears muddy-brown in nature [4,19,20]. Our patient displayed hyperpigmentation not only within scars from acne but also on his dorsal hands.

As of October 2014, databases such as the World Health Organization (WHO) and Eudraviligance (EMA) received 108 reports of discoloration due to doxycycline. They included skin discoloration (64 reports), skin hyperpigmentation (25 reports), and pigmentation disorders (19 reports) [10]. However, to the best of our knowledge and including our patient, reports providing details of the patients with doxycycline-associated hyperpigmentation have only been described in 18 individuals (Tables 1, 2) [7-16].

**Table 1:** Doxycycline-associated hyperpigmentation: epidemiology and history Abbreviations: A, age (in years); C, case; conj, conjunctivitis; CR, current report; doxy, doxycycline; F, female; G, gender; Hx, history; M, male; NS, not stated (in case report); Ref, references; surg, surgical; OOH, onset of hyperpigmentation (in months); OSM, other systemic medications. ^a^The country of origin, based on the location of the article’s authors, included the Netherlands (cases 4, 9-11, and 13-17), the United States of America (cases 6-8, 12, and 18), Turkey (cases 1-3), and Germany (case 5). ^b^Onset of hyperpigmentation is the number of months after starting treatment when the patient noticed hyperpigmentation or when the patient presented to a physician who noticed the hyperpigmentation. ^c^The patient was previously using minocycline for six years, but due to minocycline-associated hyperpigmentation of ears and fingernails, patient was switched to doxycycline 100 mg daily. ^d^The patient was receiving doxycycline 300 mg daily for six months prior to surgery, 200 mg daily for six months after surgery, and then 300 mg daily for two additional months. He received hydroxychloroquine 600 mg daily for all 14 months. He presented with hyperpigmentation after receiving 14 months of both drugs (which was eight months after surgery).

C^a^	A	G	Hx of scars	Doxy indication	Doxy dose	OSM	OOH^b^	Ref
1	11	M	NS	Brucellosis	100-200 mg daily	Yes	0.5	[7]
2	17	F	NS	Brucellosis	100 mg twice daily	Yes	0.67	[8]
3	30	F	Acne scars on face	Acne	100 mg daily	No	1	[9]
4	31 -40	F	None	Lower respiratory tract infection	100 mg daily	Yes	0.5	[10], C4
5	36	M	NS	Self-medication	Up to 1000 mg daily	NS	144	[11]
6	40	M	Acne scars	Acne	100 mg twice daily	No	0.5	CR
7	44	M	NS	Chronic follicular conj	100 mg twice daily	NS	36	[12]
8	49	M	Acne scars on face and scalp	Pustular acne induced by vandetanib	NS	NS	1.5	[13]
9	61 -70	F	NS	Respiratory infection	100 mg once to twice daily	No	0.07	[10], C3
10	61-70	M	NS	Infection	100 mg once daily	NS	0.3	[10], C5
11	71	M	NS	Chronic Q-fever	200 mg/day	Yes	37	[14], C3
12	71	M	NS	Acne vulgaris	100 mg daily	Yes	12^c^	[15]
13	71 and older	M	NS	Chronic Q-fever and infected vessel prothesis	100 mg three times daily	Yes	9	[10], C1
14	71 and older	M	NS	Chronic Q-fever	200-300 mg daily	Yes	10	[10], C2
15	72	M	NS	Whipple’s disease	200 mg daily	Yes	8	[14], C2
16	72	M	Surg scars	Chronic Q-fever	200-300 mg daily	Yes	14^d^	[14], C4
17	75	M	NS	Chronic Q-fever	200-300 mg daily	Yes	8	[14], C1
18	87	F	None	Bullous pemphigoid	200 mg daily	Yes	120	[16]

**Table 2:** Doxycycline-associated hyperpigmentation: clinical characteristics Abbreviations: C, case; CR, current report; HCQ, hydroxychloroquine; pig, pigmentation; Ref, references; TMP-SMX, trimethoprim-sulfamethoxazole ^a^None of the patients had hyperpigmentation on their mucosa (oral or conjunctiva/sclera).

C	Skin pig	Nail pig	Site of hyperpigmentation^a^	Management	Response	Ref
1	-	+	Fingernails, especially thumbs	Switched to TMP-SMX	Discoloration disappeared within one month	[7]
2	-	+	Fingernails	Discontinuation of doxycycline and rifampicin	Discoloration gets better in the next month	[8]
3	+	-	Within depressed acne scars of face	Discontinued doxycycline	Three months later, discoloration unchanged	[9]
4	+	-	Face	Discontinued	Unknown	[10], C4
5	+	-	Anterior part of lower legs	Continued self-medication, eventually hospitalized and use of doxycycline stopped	Two years later, pig patches on the lower legs had faded significantly	[11]
6	+	-	Face within acne scars, dorsal hands	Discontinued, switched to erythromycin	Recovered	CR
7	+	-	Symmetric periocular	Discontinued	Almost complete resolution at eight months after discontinuing	[12]
8	+	-	Within depressed acne scars on face and scalp	NS	NS	[13]
9	+	-	NS	Discontinued	Recovered	[10], C3
10	+	-	Face	Discontinued	Not recovered	[10], C5
11	+	-	Pretibial on both legs, dorsal side of feet	Stopped doxycycline and HCQ; substituted with moxifloxacin and rifampicin	Six months later, discoloration diminished	[14], C3
12	-	+	Proximal nail beds of both hands	NS	NS	[15]
13	+	-	NS	Discontinued	Not recovered	[10], C1
14	+	-	NS	Discontinued	Not recovered	[10], C2
15	+	-	Back of both hands	Stopped doxycycline, co-trimoxazole reintroduced	Ten months later, pig slowly facing	[14], C2
16	+	-	Around surgical scars on both legs	Stopped doxycycline and HCQ; substituted with moxifloxacin	Two months later, discoloration diminished	[14], C4
17	+	-	Lower arms, back of hands, interdigital areas	Stopped doxycycline	Twelve months later, pig slowly diminished, but macules visible on back of hands and lower arms	[14], C1
18	+	+	Diffusely on face and extremities; subungual regions	Continued doxycycline due to patient preference and benefit	Chronic discoloration remained stable	[16]

The onset age of doxycycline-associated hyperpigmentation cases ranged from 11 years to 87 years old, with a median age of between 65.5 years. This included 13 male and five female patients. The presence or absence of scars was described for six patients: two men and one woman had acne scars, one man who surgical scars on his legs, and two women did not have any scars.

Our patient was Hispanic; the racial background of the other patients was not provided. The nationality of the patient may be indicated by the origin of the manuscript. Most of the individuals (nine patients) were from the Netherlands. Thereafter, in order of decreasing frequency, the reported individuals with doxycycline-associated hyperpigmentation were from either the United States of America (five patients), Turkey (three patients), or Germany (one patient). 

The indication for doxycycline was most commonly for either managing chronic Q-fever in five patients or treating acne in four patients. Two patients were being treated with doxycycline for a nonspecific respiratory infection and the two pediatric patients were taking the medication for brucellosis. There was one patient each for treating chronic follicular conjunctivitis, bullous pemphigoid, unspecified infection, and Whipple’s disease.

There was one patient self-medicating with doxycycline. A 36-year-old man with depersonalization and derealization syndrome with recurrent raised temperatures self-medicated using oral doxycycline. He started by taking 100-mg tablets 15 to 50 times a month. He self-medicated for 12 years with an ending dose of up to 1000 mg daily. He was eventually hospitalized for impaired general condition, fatigue, and dizziness. At this visit, hyperpigmentation was noticed on the anterior part of his lower legs. After discharge, the patient continued to self-medicate for another year [11].

Doxycycline is available in either the monohydrate or hyclate form. The man in this report was receiving doxycycline monohydrate. The doxycycline formulation was not described for the other patients.

The doxycycline dose for all patients ranged from 100 mg to up to 1 g daily. Six of the patients, including the patient in this report, were taking a daily dose of 200 mg, as either a single 200 mg dose (three people) or a 100 mg dose twice each day (three people) [8,12,14,16]. Four patients were receiving 100 mg as a single daily dose [9,10,15]. The remaining patients were receiving 200-300 mg daily (three patients), 100 mg three times daily (one patient), 100-200 mg daily (one patient), or 100 mg once or twice daily (one patient) [7,10,14]. One man took up to 1,000 mg daily and the daily dose is not described in one patient [11,13].

Of the 18 patients, 11 were using another systemic medication concurrently. Hydroxychloroquine was the most common concurrently used medication, being used in five patients [10,14]. Two patients previously used another tetracycline agent before starting doxycycline [9,15].

We defined the onset of hyperpigmentation as the duration of time after starting treatment with doxycycline when either the patient or their physician noticed the pigmentary change. This ranged from two days to 12 years; the median time for onset of hyperpigmentation was eight months. Including our patient, the hyperpigmentation appeared within one or fewer months after starting doxycycline in seven patients (38.9%) and within 12 or fewer months in 13 patients (72.2%) [7-10,13-15]. Doxycycline-associated hyperpigmentation occurred in 14 months in one patient, 36 months in one patient, and 37 months in another patient [12,14]. The remaining two patients had delayed onset of hyperpigmentation at 10 years and 12 years [11,16].

In terms of hyperpigmentation type, 14 reported skin-only findings, three reported nail-only findings, and one had both skin and nail findings [7-16]. None of the patients reported mucosal involvement. Similar to minocycline-induced hyperpigmentation, the doxycycline-associated pigmentation of four of the patients, including the reported patient, had skin darkening of their scars [9,13,14,20]. 

The face was the most common site of skin hyperpigmentation presenting in seven patients, including our patient [9,10,12,16]. Four patients had hyperpigmentation on the lower limbs and four patients, including our patient, had findings on the upper limbs [14,16]. One patient had involvement of the scalp [13]. The site of skin hyperpigmentation was not discussed for three patients [10].

Skin biopsies for microscopic evaluation were evaluated in three of the patients with doxycycline-associated hyperpigmentation [9,11,12]. Two of the patients had facial pigmentation located on either acne scars or the periocular region; the pathologic findings were similar to those observed in patients with type three cutaneous minocycline pigmentation: increased melanization of the basal layer and pigment deposition in dermal melanophages [9,12,20]. The lower leg lesions of the third patient showed pathological findings similar to those observed in patients with type two minocycline-induced pigmentation; in addition to increased melanization in the basal layer of the epidermis, there were numerous pigment-laden macrophages in the dermis and the subcutaneous fat that stained positive for both melanin and iron [11,20].

The man in this report, who declined biopsy evaluation, had hyperpigmentation not only located on the face (including areas of acne scars) but also on his dorsal hands. Therefore, similar to the 30-year-old woman with doxycycline-associated hyperpigmentation in her acne scars, it is possible that his facial hyperpigmentation only showed melanization of the epidermal basal layer similar to that seen in patients with minocycline type three cutaneous pigmentation; however, the possibility of positive staining for both melanin and iron that is commonly observed in acne scar lesions of patients with type one minocycline pigmentation cannot be excluded. There are two possibilities for the pathologic findings of the reported patient’s dorsal hand hyperpigmentation: similar to patients with type two minocycline pigmentation usually observed in normal non-inflamed skin on the extremities, the changes could include positive staining for melanin and iron in the dermal pigmentation deposition; alternatively, the pathology could be consistent with that observed with type three cutaneous minocycline pigmentation occurring on sun-exposed areas and demonstrate only melanization of the basal layer and macrophages in the dermis [20].

For management, 14 of the 18 cases stopped doxycycline treatment. One patient continued doxycycline for the treatment of bullous pemphigoid and one patient continued self-medication with doxycycline; two case reports did not share details on the management of the patient. Of the 14 patients who discontinued the doxycycline, nine reported resolution of the pigment, four reported the hyperpigmentation did not resolve, and one case outcome was not described.

The time for discoloration to resolve ranged from one month to 24 months for the nine patients who reported resolution. Of the four cases that reported no resolution, one patient was reported at two days, one patient was reported at three months, and the other two patients were reported after an unknown period of time after stopping the doxycycline.

## Conclusions

Cutaneous hyperpigmentation associated with doxycycline treatment is uncommon. Including the man in this report, specific characteristics of patients with doxycycline-associated hyperpigmentation have been described in 13 males and five females. The median age of the patients was 65.5 years. The skin darkening occurred as promptly as two days to as long as 144 months after the onset of doxycycline treatment. Similar to the reported patient, some of the individuals with doxycycline-related hyperpigmentation had skin darkening that occurred in scars. Management in the majority of patients was discontinuing the doxycycline. The spontaneous resolution began as early as one month after stopping doxycycline.
